# Exploring the complex pathway of the primary health care response to intimate partner violence in New Zealand

**DOI:** 10.1186/s12961-018-0373-2

**Published:** 2018-10-19

**Authors:** Claire Gear, Elizabeth Eppel, Jane Koziol-Mclain

**Affiliations:** 1 Centre for Interdisciplinary Trauma Research, Auckland University of Technology, Private Bag 92006, Auckland, 1142 New Zealand; 2 School of Government, Victoria University of Wellington, PO Box 600, Wellington, 6140 New Zealand

**Keywords:** Intimate partner violence, Primary health care, Complexity theory, Complex adaptive system, Document analysis, Narrative, Discourse, Implementation, Policy-making, Sustainability, IPV intimate partner violence, MOH Ministry of Health, DHB District Health Board, GP General Practitioner, E Tu Whānau Programme of Action for Addressing Family Violence

## Abstract

**Background:**

Integrating sustainable responses to intimate partner violence in health care is a persistent and complex problem internationally. New Zealand holds a leading role, having established national health system infrastructure for responding to intimate partner violence within hospital and selected community settings. However, resources for, and engagement with, the primary health care sector has been limited. The present study focuses on what affects a sustainable response to intimate partner violence within New Zealand primary health care settings.

**Methods:**

Utilising complexity theory, we reconceptualised a sustainable primary health care response to intimate partner violence as a complex adaptive system. To explore interactions between agents, we analysed the function(s) of key policy, strategy, guideline and evaluation documents informing intimate partner violence responsiveness in health care. We chronologically threaded these documents together by their function(s) to show how discourse influencing intimate partner violence responsiveness emerges from agent interactions.

**Results:**

This paper presents a complexity informed implementation narrative of the New Zealand health system response to intimate partner violence across the last two decades, focused on the participation of the primary health care sector. We demonstrate how competing discourses have contributed to system gaps and unintended consequences over time. Our findings consider implications for a sustainable response to intimate partner violence in primary health care and call attention to system interactions that challenge a whole health system approach in New Zealand.

**Conclusions:**

Use of complexity theory facilitates an innovative perspective of a persistent and complex problem. Given the complexity of the problem and New Zealand’s leadership, sharing the lessons learnt is critical for the international community involved in developing health care system approaches to intimate partner violence.

## Background

Primary health care provides opportunity to disrupt the causes of ill-health, including issues that traditionally fall outside of the health sector such as intimate partner violence (IPV)^1^ [1]. Internationally, the health response to violence is now situated within a public health framework focused on preventing and mitigating the health consequences of violence [2]. Primary health care is recognised as a setting uniquely positioned to respond to those experiencing violence, being an entry point into the health system and a first, or only, point of contact with professionals who can facilitate access to specialist care and support [3]. International guidelines strongly recommend primary health care be prioritised for IPV workforce training and service delivery. Health care professionals should, at a minimum, be able to provide a first-line response to those experiencing IPV, including facilitating disclosure, offering support and referral, providing medical treatment and follow-up care, and documenting evidence [3]. However, integrating sustainable and effective responses to IPV in practice has proven challenging across health systems and settings, often being referred to as a ‘complex’ or ‘wicked’ problem [4–6]. New Zealand has been an international leader on family violence responsiveness in health care via its Violence Intervention Programme [7]. Infrastructure supporting effective responses to IPV and child abuse and neglect has successfully been implemented within hospital and selected community settings [8]. Yet, similar engagement within primary health care has been limited [5]. Utilising complexity theory, we explored what affects a sustainable response to IPV in New Zealand primary health care settings.

General practice in New Zealand is largely autonomous from public governance [9]. Under New Zealand’s policy settings, general practices receive public funding from the Ministry of Health (MOH) distributed via the District Health Board (DHB) to their regional Primary Health Organisation under service agreements. In 2016, there were 20 DHBs, 32 Primary Health Organisations and 1013 general practices [10]. Aside from funding primary health care services, DHBs are also responsible for the provision of hospital care and some public health and community services [11]. MOH funds the Violence Intervention Programme through individual contracts with each DHB [12] (for more detail on the New Zealand health system see [11]).

Complexity theory facilitates an innovative perspective of complex problems by focusing on the interaction between system elements, rather than studying them in isolation [13]. Instead of providing a prescribed methodology, complexity theory offers numerous concepts, which can be combined in different ways and alongside different theoretical models, to view complex problems in different manners [14, 15]. With increasing application in health care, complexity theory is often used to reframe health care systems as complex adaptive systems [15, 16]. Complex adaptive systems are made up of many diverse system agents (i.e. individuals or collectives involved in the system) constantly in interaction with, and adapting to, one another. Repeated patterns of agent interaction lead to spontaneous new behaviours (self-organisation) and the emergence of new system structures [17].

The complexity of IPV emerges from the entanglement of many diverse factors that contribute to, and sustain, violence in people’s lives. Similarly, the complexity of health care systems emerges from the interaction between the many diverse agents involved in health care. When these two complex systems interact, the number and diversity of interactions between agents makes it difficult to predict how things will unfold. Despite good intentions, agent interactions may generate unintended effects that challenge effective and sustainable practices [17]. Utilising complexity theory as a research methodology allows us to explore the interactions between the continuously changing inputs, relationships, outcomes and consequences involved in responding to IPV in health care settings [18]. The concept of a sustainable health care response to IPV evolves into a constantly emerging phenomenon generated by patterns of interaction between agents. This approach is fundamentally useful in calling attention to influences, known or unknown, that affect sustainable responses to IPV over time [19].

In this paper, we demonstrate the potential for a complexity-led approach to open new ways of thinking about, and responding to, complex problems. We trace the implementation pathway of the New Zealand health care system response to IPV across two decades, focused on the participation of the primary health care sector (Table 1). We demonstrate how discourse influencing IPV responsiveness emerges from agent interactions, contributing to system gaps and unintended consequences. We call attention to agent interactions that challenge the implementation and sustainability of a health system response to IPV across secondary and primary health care. Our findings are presented within a chronological narrative of implementation.

**Table 1:** Timeline *

1995	First protocol supporting general practitioner responsiveness developed and tested
1996	Government statement of policy on family violence released
1998	Ministry of Health releases first family violence guidelines (October)
1999	Death of Riri-o-te-Rangi James Whakaruru (April)
	**5th Labour Government elected (centre-left) (September)**
2000	Investigation findings into the death of Riri-o-te-Rangi James Whakaruru released (June)
	New Zealand Health Strategy released with an objective on interpersonal violence (December)
	Ministry of Health Family Violence Intervention Project commences (November)
2001	Ministry of Health releases first Primary Health Care Strategy (February)
	Ministry of Health introduces the Family Violence Intervention Project (October)
	District Health Boards established (December)
2002	Pilot testing of the Family Violence Intervention Project begins within four hospital settings (April)
	Ministry of Social Development launches first Family Violence Prevention Strategy (February)
	**Ministry of Health publishes Family Violence Intervention Guidelines (September)**
	Royal New Zealand College of General Practitioners declines to endorse the Family Violence Intervention Guidelines
2003	Royal New Zealand College of General Practitioners publishes ‘Recognising and responding to intimate partner violence’ resource (June)
2004	Centre for Interdisciplinary Trauma Research publishes baseline Family Violence Intervention Project evaluation report (November)
2005	Cross-government Taskforce for Action on Violence within Families established (June)
	Centre for interdisciplinary Trauma Research identifies gap for a primary health care response to family violence
2007	Family Violence Intervention Project concludes pilot testing
	Ministry of Health Violence Intervention Programme launched
2008	**5th National Government elected (centre-right) (November)**
	Ministry of Health funds development and pilot testing of primary health care evaluation tool (November)
2010	Ministry of Health provides Violence Intervention Programme funding to improve responsiveness to Māori
	Centre for Interdisciplinary Trauma Research makes the primary health care evaluation tool freely available (July)
2012	Centre for Interdisciplinary Trauma Research publishes primary health care evaluation tool development methods and findings, conducts a follow-up evaluation of pilot sites and hosts a national primary health care responsiveness network meeting (May)
2013	The Taskforce for Action on Violence within Families Māori Group publishes E Tu Whānau (May)
2014	Family Violence Death Review Committee publishes the Fourth Annual Report (June)
	Ministerial Group established alongside cross-government package to reduce family violence (July)
2016	Family Violence Death Review Committee publishes the Fifth Annual Report (February)
	The Royal College of New Zealand General Practitioners declines to endorse refreshed Ministry of Health guidelines (March)
	Ministry of Health publishes a revised health care strategy (April)
	Ministry of Health publishes refreshed family violence assessment and intervention guidelines (June)
	The Royal College of General Practitioners publish revised quality standards for general practice (September)
	Centre of Interdisciplinary Trauma Research publishes primary health care follow-up evaluation findings
2017	The Ministerial Group publishes two frameworks for a common and consistent approach to family violence across agencies (June)

(*Events in bold; month included where known)

## Methods

Utilising complexity theory, we viewed a sustainable response to IPV as a complex adaptive system to focus on the interaction between agents and how they communicate within the system [18]. We viewed discourse as a complex adaptive system, where dynamic patterns of agent interaction self-organise into routinised ways of interacting, generating discourse phenomena [20, 21]. Put more simply, meaning is generated by the interaction between agents. From this perspective, discourses are not static but continuously emerge from agent interactions; what may be understood in one context may mean something different in another. These discourses simultaneously shape individual and organisational values and identity and block other ways of knowing [20, 21]. For example, a practitioner who describes IPV as a problem only for low socioeconomic groups, blocks knowledge of IPV dynamics for middle and high socioeconomic groups.

A complexity-led discourse analysis allows deeper insights into how diverse agents interact to identify, define and prioritise IPV, both collectively and individually, producing a much more nuanced understanding of the discourses at play. It also calls attention to multiple opportunities to effect system change by strategically influencing agent interactions [21]. In this study, we chose to analyse documents as they provide a static representation of discourse, representing a specific understanding of an issue at a point in time [18, 22]. To access discourses, we analysed the function of key policy, strategy, guideline and evaluation documents. Focusing on the function of the documents, rather than the specific content, provides an understanding of how various system agents position and manipulate health policies shaping a system’s primary health care response to IPV [21, 23]. In this paper, rather than naming and describing discourses, we map out how discourses have self-organised into a stabilised pattern that constrains primary health care participation in the health system response to family violence. We demonstrate how numerous competing discourses have contributed to system gaps and unintended consequences over time. Further detail on our methodological approach can be found elsewhere [18].

### Data collection

Beginning with easily identifiable documents (e.g. the New Zealand Health Strategy), we applied a snowball method to source documents connected to contexts in which the document was produced. One document could lead to a variety of other documents and discourses offering an infinite view of system connectedness. We collected data to achieve saturation within the boundaries of this study. Most documents were sourced online through libraries and the Google search engine, hardcopy documents were sourced from the New Zealand Family Violence Clearinghouse Library and the National Library of New Zealand. We sourced 110 documents across three main fields, namely (1) New Zealand health care strategies, (2) international recommendations for addressing IPV in health care and (3) New Zealand family violence prevention and/or intervention documents. Selection was emergent and pragmatic, guided by the question ‘What is the relevance of the document to the research problem and purpose?’ [24]. Documents were designated as either primary or secondary material. Primary material (*n* = 33) included the most recently published version of a document directly influencing IPV responsiveness in New Zealand primary health care. Secondary material (*n* = 77), not necessarily directly relevant to IPV responsiveness, provided document context, such as document purpose(s), or further information on key issues within the primary documents such as health target critique. Secondary material included commentary, research reports, websites, media releases, discussion documents, personal communications or previous versions of the selected primary document.

### Analysis

Within each group of documents, each document was analysed to identify function(s), supported by a collation of analysis questions (informed by Rapley [22], Bowen [24], Prior [25], and Shaw, Elston and Abbott [26]), such as ‘how does the document transform our actions and interactions?’ or ‘how is the material called upon or manipulated?’, alongside supplementary knowledge from secondary materials. Working chronologically, these documents were threaded together by their function(s) within an implementation narrative. Applying the complex adaptive system analytical lens, this process called attention to how agent interactions have self-organised to shape implementation direction, contributing to system gaps and unintended consequences. These insights were written into the narrative as it emerged, generating an implementation narrative that emphasises the complexity of implementing sustainable responses to IPV within New Zealand primary health care. For the purposes of this paper, we focus our findings on the New Zealand health care response to IPV, creating an artificial boundary that excludes possible interactions between international recommendations and the New Zealand response.

## Results

The narrative is structured to call attention to the construction and use of discourses over time, interspersed with ‘events’, defined as a significant occurrence that alters the system trajectory [27, 28].

### Constructing discourse: the ‘Gardyne’ protocol

The first New Zealand protocol supporting general practitioners (GPs; primary health care doctors) to respond to IPV was developed in 1995 by an Auckland-based research team who sought to promote IPV as a significant public health policy issue [29]. The research project focused on GPs as the providers likely to first encounter signs of violence and able to provide early intervention, thereby preventing the escalation, severity and health effects of IPV [29]. The team developed the ‘Gardyne’ protocol, which alongside specialist training, provided GPs with ‘practical tools’ for communication, recognition, disclosure, safety and referral across two interventions, namely to women (as victims) and men (as perpetrators) [30]. The protocol was tested with 25 Auckland-based GPs but no evaluation of effectiveness was published [29]. The team found that, unlike other issues (such as alcohol abuse), there were no resources for responding to IPV and many GPs were concerned they were working ineffectively by missing cases of IPV or intervening poorly [29]. Interim recommendations included disseminating and implementing the protocol within health services and over time establishing a dedicated health care service for victims of IPV [29]. This research project may represent the initial construction of discourse phenomena around GP responsiveness to IPV in New Zealand. The intent to integrate the protocol within health services may be understood as a first attempt to influence primary health responsiveness to IPV from the ‘bottom-up’ (i.e. GP developed). However, the impact of this research remains unclear, suggesting interactions with other health system agents were blocked.

### Formalising discourse: establishing policy and guidelines

In 1996, New Zealand released a Government Statement of Policy on Family Violence, actioning the 1994 New Zealand Crime Prevention Strategy priority to reduce the incidence of family violence [31]. The Statement functioned to set a common policy approach for all government agencies involved in responding to family violence [32, 33]. As intended, the Statement principles formed the core of the health system response to family violence, adapted by the MOH for the first government-led health care family violence guidelines published in 1998 [32, 34].

Prior to the 1998 guidelines, health care responses had been ad hoc and largely focused on child abuse and neglect [34]. The 1998 guidelines represented a development phase of the health system response to family violence, functioning as a first policy step toward coordinated and consistent responses [34]. Developed by the MOH Public Health Group, the guidelines define the health sector role as responding to the adverse health effects of violence through prevention and crisis intervention. The role is framed by the WHO Ottawa Charter, improving public health by raising awareness of violence, fostering non-violent behaviour as well as access to medical help and a safe environment [32, 34, 35]. The 1998 guidelines were designed to support health care providers to develop family violence protocols in their local setting, facilitating consistency across the sector while assigning responsibility for development and use of protocols to providers. The MOH was responsible for disseminating the guidelines and encouraging their use through provider training and contract quality requirements [32, 34]. Primary health care was identified as one priority setting for initial protocol development and training, referencing the initial ‘Gardyne’ protocol developed in 1995 [30, 34]. Defining family violence responsiveness within policy and guidelines set the knowledge boundaries of how health care may respond to IPV (i.e. addressing a public health problem in a consistent manner), establishing a pathway from which future agent interactions would be influenced. However, the strategically planned ‘top-down’ method of implementation was to be challenged by two significant events, namely the introduction of the primary health care strategy and the death of Riri-o-te-Rangi (James) Whakaruru.

### Event: the Primary Health Care Strategy 2001

In 1999, a newly elected government initiated significant health system reform through the New Zealand Health Strategy 2000 and Primary Health Care Strategy 2001 [36, 37]. This was the first time the government had set a vision for how primary health care would be organised and delivered [38]. Key to the new strategy was a population health approach that organised service delivery around the needs of defined populations, rather than responding only to those who actively sought care. Capitation funding was based on the characteristics of enrolled populations, allowing greater flexibility in service utilisation and reduced costs for patients [36, 38].

The strategy established a new layer of organisation, including Primary Health Care Organisations and Māori^2^ Development Organisations, designed to provide a central point of contact for both community and secondary care providers. This intermediate organisational layer was tasked with improving the health of their population by responding to the Health Strategy objectives [39, 40]. Only GPs who joined Primary Health Organisations or Māori Development Organisations were eligible for the new population-based funding. This strategy functioned to make all primary health care professionals responsible for meeting the needs of their populations, reducing the medical dominance of one professional group (such as GPs) [36, 39].

### Event: the death of Riri-o-te-Rangi (James) Whakaruru

During the 2000s’ health system reform, development of the health system response to family violence was accelerated by the death of 4-year-old James Whakaruru on April 4, 1999, from one or more physical assaults by his mother’s partner [41]. His death sparked an investigation that found poor communication between statutory agencies had failed to protect James [42]. The health sector had had extensive contact with James, but failed to communicate the necessary information between practitioners:

“*James was seen forty times by health practitioners, four presentations at the hospital emergency department, two admissions and one outpatient clinic, three face-to-face Plunket* [child health provider] *contacts, and thirty visits to general practitioners at four practices. Collectively the health sector had available a telling picture of James’ circumstances. This picture was never put together because of poor communication between practitioners*” ([42], p. 4).

The investigation report made 59 recommendations to be implemented by government agencies [43, 44]. The MOH issued a report detailing the health sector response to the findings [41]. Among others, actions included establishing a priority objective within the New Zealand Health Strategy 2000 to “*reduce the incidence and impact of violence in interpersonal relationships, families, schools and communities*” ([37], p.vii). This objective was important as it directed the health sector to focus on actions to increase family violence responsiveness. It also set an expectation that newly formed DHBs (established in 2001) would implement family violence programmes based on the guidelines [45].

James’ death and the investigation recommendations directed the focus of action toward responding to child abuse and neglect in hospital settings, unintentionally suspending action in primary health care. The 1998 guidelines were to be implemented, hospital-based policies on child abuse management reviewed, a national policy on the use of skeletal surveys for non-accidental injury implemented, and national child abuse and neglect guidelines and training developed. Notably, the report observed the difficulty of GPs in gaining oversight of James’ social circumstances; this appeared unaddressed by the Ministry [41].

The events stemming from James’s death forced health-system agents to co-evolve. The Health Strategy objective shifted the responsibility of implementing a response from individual health care providers to the level of DHBs. The recommendations held the MOH responsible for developing national child abuse and neglect guidelines. At the same time, the introduction of the Health Strategy and Primary Health Care Strategy made health providers responsible for reducing the incidence and impact of interpersonal violence within their populations while initiating significant organisational reform across the sector, particularly within primary health care [36, 37]. It can be argued that the simultaneous introduction of the Health Strategy objective and organisational reform had a negative influence on the uptake of family violence responsiveness in primary health care, an unintended consequence.

### Introducing discourse: the Violence Intervention Project

To support DHBs in actioning the Health Strategy objective, the MOH published a Toolkit in 2001 suggesting how the health sector could respond to “*interpersonal violence*” ([46], p. 4). Similar to the 1998 guidelines, the Toolkit was framed by public health noting that interpersonal violence is common, associated with significant health effects and high health care utilisation and costs [45, 46]. The Toolkit encouraged DHBs to reduce interpersonal violence through the use of population-based strategies (i.e. health promotion activities) and family violence interventions to identify, assess and refer those experiencing violence [45, 46]. Again, primary health care was identified as one of ten target services where disproportionate numbers of people experiencing family violence may present [46]. The Toolkit also promoted creating institutional change by adopting a systems approach to strengthen health care responses to family violence [46]. As such, it introduced the MOH Family Violence Intervention Project as the focus of the health sector response to interpersonal violence. With the introduction of the Violence Intervention Project, the health system response moved into an implementation phase. The Project aimed to develop the 1998 guidelines alongside three major objectives of (1) establishing practice procedures (or protocols) to identify, assess and refer victims of family violence, (2) funding health professional training and (3) piloting DHB implementation of the 1998 guidelines. This work was supported intersectorally by the launch of the first New Zealand Family Violence Prevention Strategy ‘Te Rito’ by the Ministry of Social Development in 2002 [47, 48], and later a cross-government Taskforce for Action on Violence within Families established in 2005 [49]. This progress was reinforced by the publication of family violence intervention guidelines.

### Event: new guidelines

In 2002, the Family Violence Intervention Guidelines: Child and Partner Abuse (2002 guidelines) were published, fulfilling the first objective of the Violence Intervention Project and the recommendations for national child abuse and neglect guidelines and training [41, 50]. The 2002 guidelines are described as a practical tool for assisting health professionals to identify and respond to family violence through a six-step model in conjunction with ‘train-the-trainer’ workshops, facilitated by the Violence Intervention Project, fulfilling the second objective of the Violence Intervention Project [45, 50]. Written generically, the 2002 guidelines were designed to be applicable to diverse health care professions and settings with an expectation of profession-specific adaptations in due course [50].

The 2002 guidelines functioned as a fundamental part of the health system response to family violence. Along with the Health Strategy objective, it was expected that all DHBs would work towards implementing the guidelines [45, 51]. Internationally, health care responses historically focused on addressing child abuse and neglect. Uniquely, the 2002 guidelines extended the focus of the recommendations by recognising the high co-occurrence between IPV and child abuse and neglect, seeking to guide an integrated response [51]. ‘Refreshed’ guidelines were published in 2016, aligning with updated policy, research and practice information [7, 52]. Rather than being adaptable to different settings, the refreshed guidelines strongly advocated for a ‘whole of system’ approach to family violence intervention and assessment. They note the growing efforts to address family violence in primary health care and place ‘increased emphasis’ on planning care transitions, such as between secondary and primary health care ([7], p. 1). Nevertheless, primary health care professionals considered the guidelines not applicable to primary health care settings. It could be argued that this stance emerged from agent interactions following James’ death, which directed action toward hospital settings and unintentionally suspended action in primary health care.

### Competing discourse: GP dissent

In 2002, the guidelines were endorsed by a number of health and social organisations, but notably excluded the Royal New Zealand College of General Practitioners, a leading professional body of GPs [50]. The Royal New Zealand College of General Practitioners declined to endorse both the 2002 and the refreshed 2016 guidelines due to a concern that:

“*The Guideline is aimed at secondary care which sees only a small portion of those with family violence as an issue, and where doctors are largely uninvolved in programme implementation.* […] *We know that 80% of women and families are seen in general practice every year, and primary care doctors and nurses have the skills and opportunity to routinely enquire in the context of a safe and trusted environment and relationship*” ([52], p. 26).

In 2003, the Royal New Zealand College of General Practitioners led the publication of a general practice ‘resource’ for responding to IPV [53, 54]. Although not indicated in print, the resource was adapted from the 2002 guidelines to be general practice relevant, providing a practical toolkit of knowledge and skills to support IPV responses in practice, alongside training (Healy C, Personal communication). As such, the resource functions as an educational point of reference for responding to victims of violence, rather than a protocol. The MOH contracted Medical Sexual Assault Clinicians Aotearoa (an expert body in sexual assault/abuse medicine) to deliver training to GPs and practice nurses, referencing both the 2002 guidelines and the general practice resource [55]. However, due to a lack of supporting infrastructure (e.g. a dissemination strategy), delivery of training was limited to interested primary health care audiences (Healy C, Personal communication). In contrast, hospital settings received ongoing nationally standardised training sessions, which increasingly became mandatory for DHB clinicians following the launch of the Violence Intervention Programme in 2007 [56]. The lack of endorsement of what became a foundational piece of the health system response to family violence served to further limit the participation of primary health care as the health system response moved into an implementation phase.

### Reinforcing discourse: the Violence Intervention Programme

The Violence Intervention Project was pilot tested in four hospital settings, between 2002 and 2007, fulfilling the third implementation objective set in 2001. The pilot sites were selected based on the involvement of those who were championing the Violence Intervention Project to date (including the DHB cited in the death of James Whakaruru) (Koziol-McLain J, Personal communication). This method created a significant, though unintended, gap as no primary health care pilot sites were included [45]. In 2007, following significant progress by the pilot sites, the Violence Intervention Project was formally launched by the MOH as the Violence Intervention Programme supported by Vote Health funding [56–58]. Following the DHB Toolkit, the Violence Intervention Programme was premised on a standardised systems approach seeking to *“reduce and prevent the health impacts of violence and abuse through early identification, assessment and referral of victims presenting to health services*” ([56], p. 1). Implementation of the 2002 guidelines (not endorsed by the Royal New Zealand College of General Practitioners) was central to the programme, supported by nationally standardised training for hospital settings, DHB family violence coordinators, resources, technical advice and national networking [59]. Uniquely, the MOH commissioned a comprehensive external longitudinal evaluation of the Violence Intervention Programme, led by the Auckland University of Technology Centre of Interdisciplinary Trauma Research [51]. Evaluation reports functioned to provide DHBs and the MOH detailed implementation information nationally, significantly contributing to the direction of the health system response to family violence [7]. However, despite the Violence Intervention Programme aim of work across DHBs (inclusive of primary health care settings), service delivery and evaluation was contracted to six target settings, namely emergency, child health, maternity, sexual health, mental health, and alcohol and drug abuse [59]. This directed the focus of implementation to those services, creating a gap in other services such as primary health care. The continued use of the term DHB served to obscure the absence of work occurring in primary health care settings (Healy C, Personal communication).

The Violence Intervention Programme evaluation was also limited in measuring work within primary health care. The partner abuse audit tool used to measure implementation was modified from a United States tool designed to measure hospital-based domestic violence programmes, which did not include indicators for primary health care [60, 61]. While early evaluation reports reflect the Violence Intervention Programme’s intention to include GPs in training [45, 62], further information on this work is not given in reports from 2007 onward, suggesting a lack of engagement from either the Violence Intervention Programme, GPs or both. Nevertheless, evaluation reports consistently noted the need to include primary health care settings to achieve family violence prevention targets [8, 45, 56, 58, 59, 62]. In 2012, it was noted that primary health care family violence programmes were being introduced opportunistically in some DHB regions [5, 8]. However, the contract for and design of the Violence Intervention Programme evaluation prevented this work being captured, limiting an understanding of responses to family violence within the primary health care sector. Primary health care participation in the health system response was further challenged with the election of a new government.

### Event: a change in political ideology

In 2008, the population health approach was diluted by a newly elected government that sought to improve health service performance through an ‘investment approach’. Applied more widely than health care, the investment approach uses data to decide which public services provide longer term returns on investment [63]. For health care, the investment approach uses data to fund services that perform well with the rationale that we may then better respond to high-need populations to avert even higher long-term costs [64]. This created a heavy focus on achieving a select few health system targets instead of enabling agents to identify and respond to population needs and risks [65]. The investment approach seriously limited the ability of general practice to innovate responses to health issues beyond the target foci [5]. The shift to the investment approach was heavily criticised as short-sighted [65, 66], as a weak population health focus unintentionally marginalises primary health care (essential to delivering population health), increasing potential for health inequity [67, 68]. As responding to family violence was not a health target, the new performance approach to health care undervalued and indirectly undermined primary health care agent interactions seeking to progress responsiveness.

### Competing discourse: supporting a primary health care family violence response

The gap in knowledge and support for a primary health care response was initially identified in 2005 [69]. In 2008, the MOH provided Violence Intervention Programme evaluation funding to develop an evaluation tool to guide family violence responsiveness in primary health care settings [70, 71]. The Centre of Interdisciplinary Trauma Research modified a United States primary health care quality assessment tool for the New Zealand context and piloted it within six volunteer primary health care sites [71, 72]. Following the Violence Intervention Programme, the tool advocated for a systems approach to support primary health care settings in implementing family violence intervention practices [71]. The tool was made freely available, though no resources to support implementation (i.e. funding) were provided and dissemination to primary health care audiences was limited.

In 2012, capitalising on building momentum in the sector and the evaluation tool, the Centre of Interdisciplinary Trauma Research, utilising additional funds provided by the MOH, hosted a meeting for primary health care professionals interested in developing a formal response to family violence. Delegates formed a National Network that developed five recommendations to progress family violence responsiveness in primary health care. The report of the meeting emphasised a critical need to support the growing momentum of primary health care professionals responding to those experiencing family violence. However, a government-directed focus on specific health targets, a lack of MOH funding and appointed leadership for responding to family violence within primary health care and no linkage to policy advocacy within the MOH, meant the report remained unpublished, limiting its influence within the sector.

Alongside the National Network, the Centre of Interdisciplinary Trauma Research led a follow-up evaluation of three of the six original pilot sites in 2012 [5]. Given the limited understanding of how to integrate responses to family violence within health care systems internationally, the published findings shared the experience of pilot site development, demonstrating the challenge of implementing a complex intervention within a complex setting. Notably, each of the pilot sites had successfully acquired fixed-term funding to support response development [5]. The paper strongly supported a systems approach to family violence responsiveness, shaped by the use of the evaluation tool as well as the simultaneous effective implementation of the Violence Intervention Programme. It proposed the use of complexity theory to explore why quality improvement methods (i.e. the evaluation tool) may effect minimal change [5]. Nevertheless, the new investment approach to health care blocked the progress of these initiatives.

### Competing discourse: responsiveness to Māori

In 2013, the Māori Reference Group for the Taskforce for Action on Violence within Families [73, 74] published the second E Tu Whānau: Programme of Action for Addressing Family Violence (E Tu Whānau). E Tu Whānau is a key policy document addressing issues of violence for Māori who, as a colonised population, are over-represented in poor social and health outcomes, including family violence prevalence and deaths [75]. E Tu Whānau provides a framework for government and Māori to work together to improve outcomes for Māori over a 5-year period. As a guiding document, E Tu Whānau functions to articulate and formalise the belief that Māori can successfully address violence within Whānau utilising Māori strengths, opening space for Māori to lead design and implementation of their own solutions to violence [74, 76, 77]. In 2010, the MOH provided additional funding and resources to improve Violence Intervention Programme responsiveness to Māori [59, 78]. However, we did not find an indication of interaction between E Tu Whānau and the Violence Intervention Programme within policy or strategy documents. How E Tu Whānau shapes policy and practice for family violence responsiveness within primary health care remains to be seen.

### Constructing discourse: reframing the approach

In 2014, a cross-government package to reduce family violence and a Ministerial Group on Family Violence and Sexual Violence were established [79, 80]. The Ministerial Group was tasked with leading a work programme to *“achieve an integrated system for preventing and responding to family and sexual violence*” ([80], p. 3) involving all agencies, led by Ministers of Justice and Social Development. Concurrently, the Family Violence Death Review Committee, tasked with investigating how to reduce the number of family violence deaths, published their fourth annual report [81]. Alongside other recommendations, the fourth report added to the momentum in the primary health care sector, specifically highlighting GPs as a consistent and frequent point of contact for families over time and recommending GPs as one of three professional groups in need of education and training. The report also encouraged the extension of the Violence Intervention Programme within primary health care [81]. Drawing on this support, the Royal New Zealand College of General Practitioners cited the Family Violence Death Review Committee report as evidence in their decision to decline endorsement of the 2016 guidelines [52]. At the same time, the Family Violence Death Review Committee published their fifth report proposing a new ‘Integrated Safety System’ recommending a nationally funded systems approach to the Violence Intervention Programme within primary health care [82]. Notably, the Family Violence Death Review Committee’s work was not reflected within the ‘refreshed’ Health Care Strategy (2016), which does not specifically address family violence [64, 83]. It was also not reflected in the revised 2016 Royal New Zealand College of General Practitioners quality standards for general practice. Participating in “*health sector family violence programmes*” is included in the standards as an “*advanced and aspirational-only indicator*” that high-performing general practices may use to voluntarily develop their services ([84], p. 170).

In 2017, the Ministerial Group published a Family Violence Risk Assessment and Management Framework alongside a Family Violence, Sexual Violence and Violence within Whānau: Workforce Capability Framework [85, 86]. These documents seek to establish a common and consistent approach to family violence across all agencies, services and practitioners as well as a minimum base level of provider knowledge, skills and behaviour needed to respond effectively to those experiencing violence. The documents position health care providers as a ‘generalist agency’, which, as a ‘primary responder’, is tasked with identifying or responding to a disclosure of family violence and facilitating access to services who can help. Notably, the Risk Assessment and Management Framework only requires health professionals to identify and refer family violence, and excludes risk assessment and safety planning, arguably a large practice gap in an effective and sustainable primary health care response [19, 87]. When consulted on the development of the Risk Assessment and Management Framework in 2016, The Royal New Zealand College of General Practitioners advocated that “*the health sector should be leading (or at least much more involved in) this work, and that GPs must be included throughout its continued development*” [88]. Fortunately, the Ministerial frameworks are considered foundational and are intended to be adapted over time [85, 86]. One could argue that the intent of these documents to implement a common and consistent approach across agencies is the most prominent sign of progress on the pathway initiated by the 1996 Government Statement of Policy on Family Violence and the 1998 guidelines. Despite efforts to redirect the system to be more responsive, the minimal inclusion of health care in the cross-government package to reduce family violence suggests patterns of interaction between agents have not shifted sufficiently to allow emergence of a dominant discourse promoting health care responsiveness to family violence, particularly within the primary health care sector. Strongly competing discourses function to block sustainable and effective primary health care responses to IPV.

## Discussion

Integrating an effective and sustainable response to IPV has proven a persistent and complex problem for health systems and settings internationally [4, 5]. Over the last two decades primary health care in New Zealand has consistently been identified as a priority setting where disproportionate numbers of people experiencing IPV may present. Yet, the sector continues to be under-utilised in the work to reduce family violence, diminishing the potential for a whole of health system approach to family violence. Complexity theory has enabled us to explore what affects a sustainable response to IPV within New Zealand’s primary health care settings. Reconceptualising the research problem as a complex adaptive system calls attention to how interaction between system agents with these documents leads to the emergence of discourse influencing IPV responsiveness. We analysed the function(s) of different policy, strategy, guideline and evaluation documents to map out how these patterns of interaction have self-organised in a way that limits the participation of primary health care in the health system response to IPV. Our analysis emphasised system gaps, unintended consequences and implications for establishing a whole system approach to family violence across both secondary and primary health care.

In particular, we call attention to three system interactions that are currently challenging a sustainable response to IPV in primary health care. First, health care responses to IPV are consistently situated within a public health approach, tasked with preventing the adverse health effects of violence [2]. However, since the Health Strategy 2000, IPV has not been recognised as a determinant of ill-health within key documents that guide health care service delivery [37]. The absence of the Violence Intervention Programme within primary health care amplifies this gap for primary health care professionals. This paper illustrates the lack of consistency across system agents in recognising IPV as a key determinant of ill-health over time.

Second, and related to the first, is the absence of a policy directive requiring primary health care professionals to respond to IPV as a determinant of ill-health. Political commitment and leadership of the issue is necessary to ensure meaningful change, adequate funding and system coordination [4, 5, 89]. This paper illustrates how shifts in political ideology, e.g. from population-based health to an investment approach, curbed agent ability to respond to health issues outside of health target foci. It also curbed political and policy leadership of the issue, stalling use of the evaluation tool and the momentum of the National Network. Recognition of IPV as a determinant of ill-health is needed within health policy despite health system governance preferences.

Third, there is a lack of engagement at both organisational (such as the Royal New Zealand College of General Practitioners) and individual GP and practice nurse levels. New Zealand GPs hold a unique position in the health system, independent of public governance. This means that, despite political leadership, GPs are able to circumvent system hierarchy by adapting policy directives through implementation [40]. This paper illustrates the ongoing GP opposition to MOH guidelines and associated training deemed inappropriate for, or unendorsed by, primary health care. Yet, by omission, the analysis also indicates a lack of response by the MOH to address these issues, and its consequent dampening effect on primary health care participation in the health-system response to family violence. Active MOH engagement with primary health care professionals appears to be needed to understand how responding to IPV in primary health care occurs, along with GP engagement to promote IPV as a determinant of ill-health.

This paper applied an innovative methodology to facilitate new understandings of a persistent and complex problem. However, our dataset was limited by its focus on documents that directly influenced IPV responsiveness in health care, omitting wider influences such as community responsiveness or gender equality. Further, analysis of interactions between agents was limited by a largely static view of the complexity provided by documents we selected within our study boundaries. Although complexity theory is useful in eliciting the complexities of the problem, it also means that interpretation will vary depending on the context in which they are read. We sought to call attention to agent interactions to open discussion on what they mean and how we might manipulate them to increase IPV responsiveness. Our next steps are to analyse interview data from front-line primary health care professionals on what occurs in practice. Combining these data sources will provide rich and diverse data in which we may explore and test for agent interaction pattern consistencies and inconsistencies that challenge or promote sustainable responses to IPV.

## Conclusions

Our use of complexity theory contributes an innovative perspective of an internationally complex problem. Yet, this is only one part of the complexity involved in implementing sustainable health care responses to IPV. Our implementation narrative exemplified the nature of sustainability as continuously emerging from the interaction between system agents, known or unknown. However, our analysis called attention to three system interactions critical to engaging the whole health system in responding to IPV. There is potential to intervene in these interactions to nudge the system in the desired direction, i.e. address IPV as a determinant of ill-health, establish a policy directive to respond to IPV, and engage with the primary health care sector to promote IPV as a determinant of ill-health. New Zealand holds a leading international role on responding to family violence in health care. Given the complexity of developing and implementing sustainable health care responses to IPV, this paper contributes valuable insights for the international health care community involved in responding to IPV.

## Footnotes

1 IPV is but one part of violence within familial relationships that we refer to as family violence.

2 Indigenous people of New Zealand
